# The discovery and significance of pegmatite–type high–purity quartz deposits in the Shangnan of North Qinling, China

**DOI:** 10.1371/journal.pone.0354364

**Published:** 2026-08-19

**Authors:** Qingqing Kang, Zhuang Min, Lei Li

**Affiliations:** 1 Jiangxi Provincial Key Laboratory of Genesis and Prospect for Strategic Minerals, East China University of Technology, Nanchang, Jiangxi, China; 2 Team 224 of Sino Shaanxi Nuclear Industry Group, Xi’an, Shaanxi, China; 3 College of Water Conservancy, Jiangxi University of Water Resources and Electric Power, Nanchang, Jiangxi, China; Tulane University School of Medicine, UNITED STATES OF AMERICA

## Abstract

High–purity quartz is a critical strategic mineral resource for high-tech industries. While exploration of granitic pegmatite-type deposits has advanced in areas like the Altai and East Qinling Mountains, key scientific questions remain regarding the dense vein-type quartz deposits within Caledonian pegmatites of the East Qinling region. Specifically, it is unclear which types of pegmatites host high-purity quartz, what processes control quartz purity, and how mineralization differs from other pegmatite quartz systems. To address these questions, we conducted field surveys, detailed studies of mineralized veins, and geochemical analyses. The results show that high-purity quartz is primarily hosted in highly fractionated Li–Cs–Ta-type pegmatites, where intense albitization and late-stage fluid activity effectively removed impurity elements. Comparative analysis indicates that vein structure, mineral assemblages, and fluid evolution pathways differ significantly between mineralized and barren pegmatites. This study clarifies the metallogenic mechanism of dense vein-type high-purity quartz deposits and provides criteria for future exploration in the region.

## 1. Introduction

High–purity quartz (HPQ), typically defined as natural quartz containing less than 50 μg/g of total impurity element content [1,2], is an indispensable strategic raw material for high-tech industries [3,4]. Its exceptional physical and chemical properties make it irreplaceable in the manufacturing of semiconductors, photovoltaic cells, optical fibers, and precision optics [5], natural sources of HPQ raw materials include crystal quartz, quartz vein, and pegmatites, with pegmatites serving as the primary global source for HPQ production [6]. In recent years, significant progress in the exploration of HPQ raw materials has also been achieved in granitic pegmatites in regions such as the Altai and the East Qinling in China [7–9]. The East Qinling region hosts numerous dense pegmatite zones associated with Caledonian plutons, including Guanpo, Longquanping, Luanzhuang, and Shangnan [10]. These pegmatite zones are known to contain pegmatite–hosted deposits such as uranium, rare metals, and others, including the Guangshigou pegmatite–hosted uranium deposit, the Xiaohuacha pegmatite–hosted uranium deposit, and the Fenghuangzhai pegmatite–hosted lithium–beryllium deposit.

In recent years, institutions have discovered pegmatite–hosted HPQ deposits in the Longquanping area. Quartz purification tests have achieved purities exceeding 99.995% [7,11], providing critical insights into the exploration of high–purity quartz deposits in the East Qinling. These findings highlight the region's substantial resource potential. Recent field investigations in the Shangnan area of Shaanxi Province have identified multiple pegmatite veins with promising high-purity quartz mineralization potential during our rare-metal pegmatite exploration [12]. Purification experiments conducted on several large–scale pegmatite veins demonstrated exceptional results, with quartz purity consistently exceeding 99.99%. Notably, a significant proportion of samples reached purity levels above 99.995%, with exceptional cases achieving 99.997% or higher. The high density of these mineralized pegmatite veins suggests considerable exploration potential for HPQ resources in the region [12]. However, current knowledge remains largely descriptive. A critical scientific gap exists in understanding the specific genetic processes controlling HPQ formation within these Caledonian pegmatites [13].

To address these questions, this study combines field geological investigations with systematic mineralogical and geochemical analyses of mineralized veins, incorporating a comparative assessment of diverse metallogenic pegmatite types. The integrated approach reveals diagnostic features of high-purity quartz mineralization, offering new insights for both theoretical understanding and practical exploration of HPQ deposits.

## 2. Regional geological setting

The research area is located at the eastern part of the Northern Qinling Orogen, within the active continental margin arc of the Northern Qinling, situated between the Shangdan suture zone and the Caichuan fault. The area has experienced multiple phases of tectonic activity [13]. The Proterozoic Qinling group (Pt_1_*qn*) dominates the area, comprising a suite of deep metamorphic rocks including gneiss, amphibolite, and migmatite. The Proterozoic Danfeng group (Pz_1_*dn*), distributed in the southwestern part of the research area, is a suite of mafic volcanic rocks [8]. Its lower sequence consists of clastic rocks interbedded with mafic pillow lavas, while the upper sequence is composed of ultramafic–mafic to intermediate–acidic volcanic rocks, which are faulted against the underlying Qinling Group. Meso–Cenozoic strata, primarily clastic rocks, are mainly distributed in the northeastern part of the research area. Various magmatic rocks are well–developed in the region, particularly Caledonian granites and granitic pegmatites [9,14]. The current study focuses on the Shangnan pegmatite concentrated zone, one of the four major pegmatite–dense zones surrounding the Huichizi pluton.

**Fig 1:**
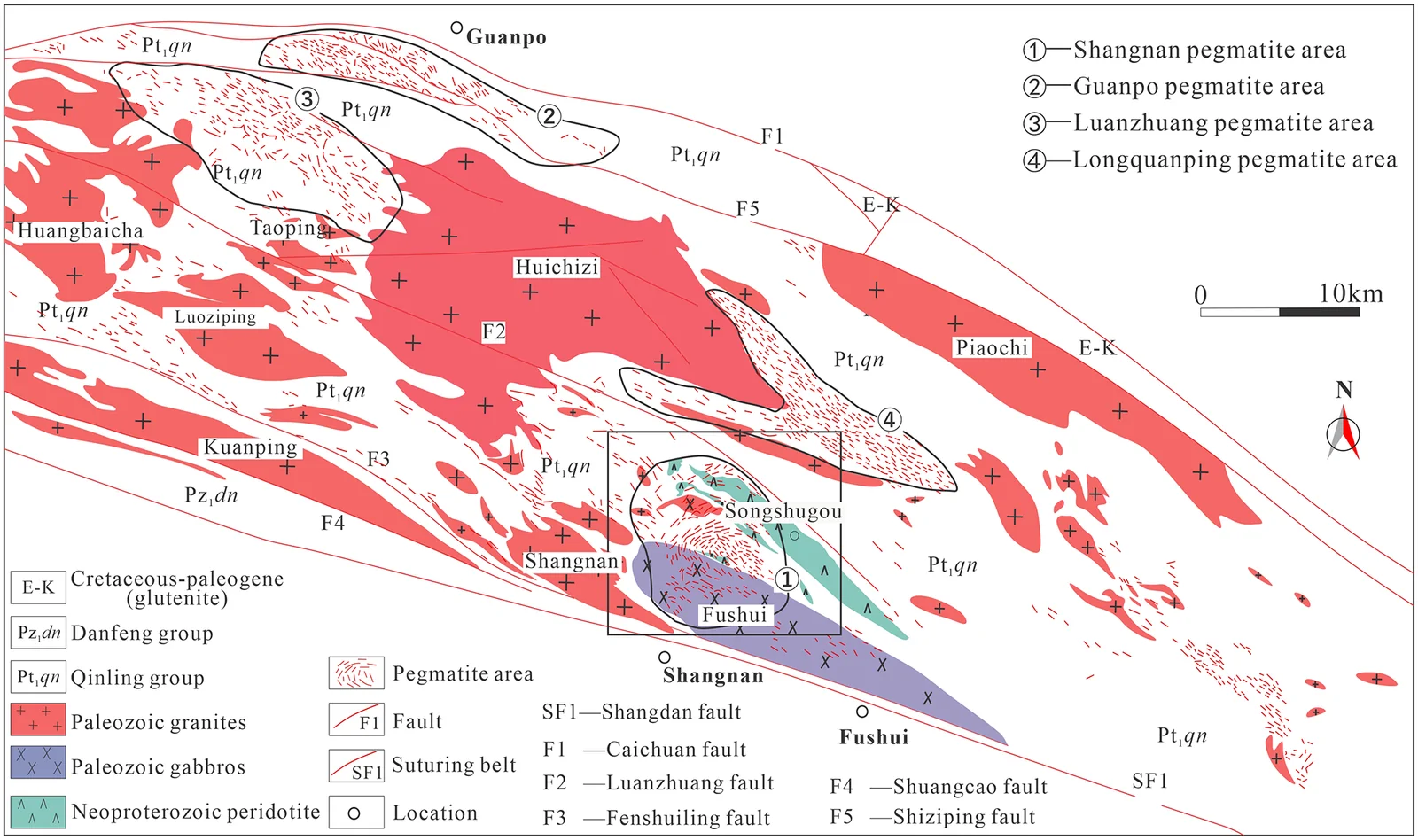
The geological structure and the distribution of pegmatite veins in the eastern Qinling (adapted from [15]).

The Shangnan Pegmatite zone is situated between the Luanzhuang fault and Fenshuiling fault. From north to south, it features the Songshugou ultramafic complex, the Qinling group (Pt_1_*qn*) gneisses, and the Fushui intermediate–mafic complex (Fig 1). This pegmatite zone covers an area of approximately 30 km^2^ and contains over 1,000 pegmatite veins. These veins typically range in length from 20 to 1500 m and in thickness from 0.5 to 50 m. The morphology of the pegmatite veins varies significantly, often exhibiting branching and composite characteristics in their strike and dip, or forming irregular, locally swollen pod–like shapes. On a planar scale, the pegmatite veins are distributed in a semi–annular pattern with an open end oriented to the southeast (Fig 1). The dip directions of the veins radiate outward from the center of the semi–annular structure. For instance, the pegmatites primarily investigated in this study are located in the northwestern segment of the semi–annular distribution, where most veins dip northwest or north. The dip angles of the pegmatite veins vary greatly, with some veins nearly vertical, while others are nearly horizontal, as exemplified by the π15 lithium–mineralized pegmatite vein at Fenghuangzhai [12].

Previous studies have classified pegmatites in the region based on the compositional characteristics of primary minerals, such as mica and feldspar, as well as their differentiation degree (structure) [9,16]. According to mineral composition, the pegmatites are divided into seven types: biotite microcline, two–mica microcline, muscovite microcline albite, muscovite albite, lepidolite microcline albite, and lepidolite albite types [9]. All these types are represented within the research area. Based on the degree of differentiation (structure), pegmatites are categorized into five structural types: fine grained, medium–fine grained, coarse grained, massive, and fully differentiated pegmatites [16]. These structural types frequently coexist within the same pegmatite vein, forming the characteristic structural zonation typical of pegmatites.

## 3. Geological characteristics of study area

In this study, nearly 50 large–scale pegmatite veins with potential for HPQ mineralization were selected within the Shangnan concentrated zone. Detailed investigations were conducted primarily on six pegmatite veins located in the relatively concentrated northwestern part of the study area, where quartz purification to over 99.99% has been achieved.

**Fig 2:**
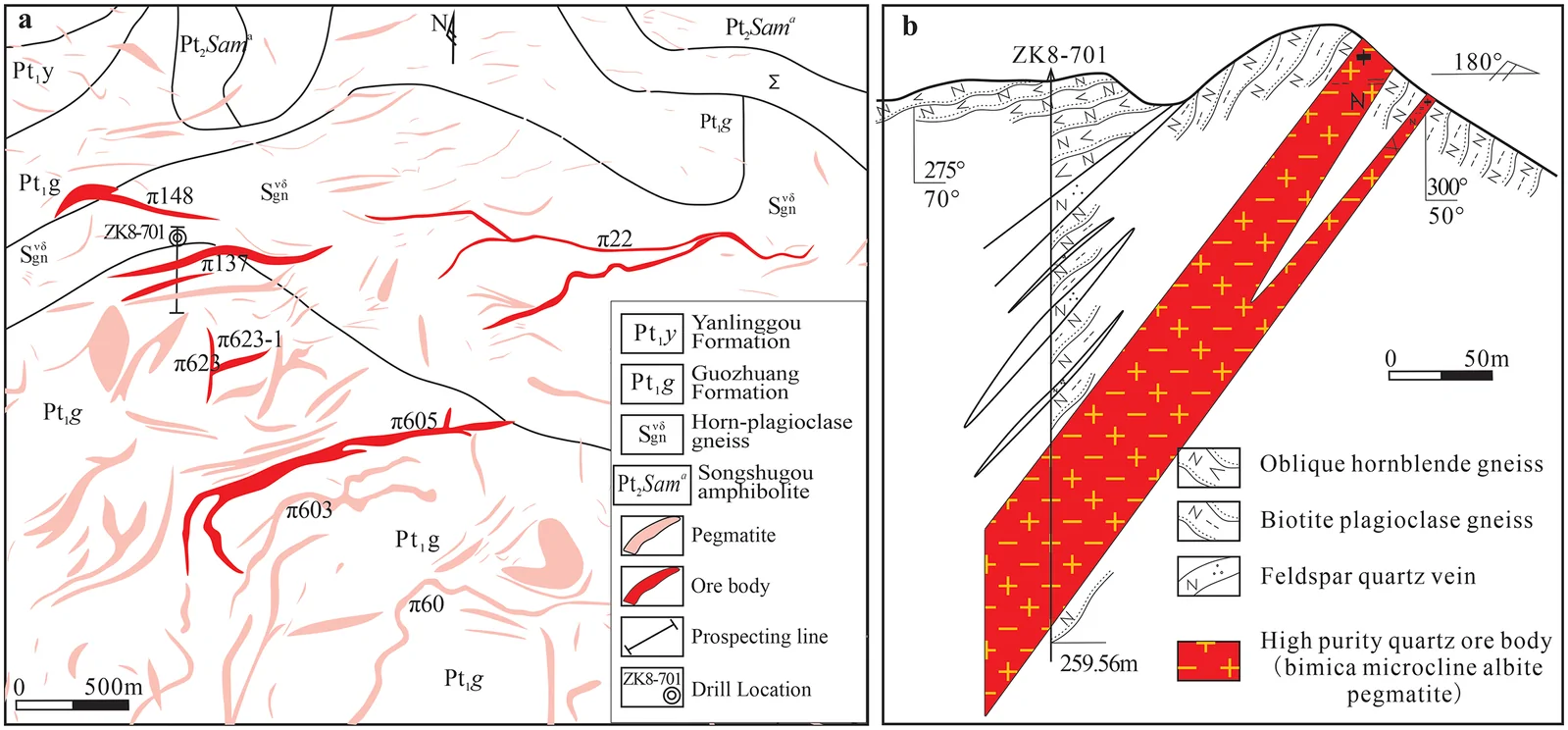
Geological sketch of HPQ deposit in Fenghuangzhai area of Shangnan (a) and profile of No.8–7 exploration line (b).

The pegmatites are mainly classified as two–mica microcline type and two–mica microcline–albite type. The veins typically range in length from 500 to 1,500 m and in width from 10 to 70 m. They exhibit a sharp intrusive contact relationship with the surrounding gneiss. Expansion, contraction, and branching phenomena are observed along their strike and dip (Fig 2). The primary mineral components of the pegmatites are microcline, albite, and quartz, with accessory minerals including muscovite, biotite, and garnet. Quartz content accounts for approximately 15–25%, with significant variability in mineral composition and content across different structural zones within the pegmatites.

**Fig 3:**
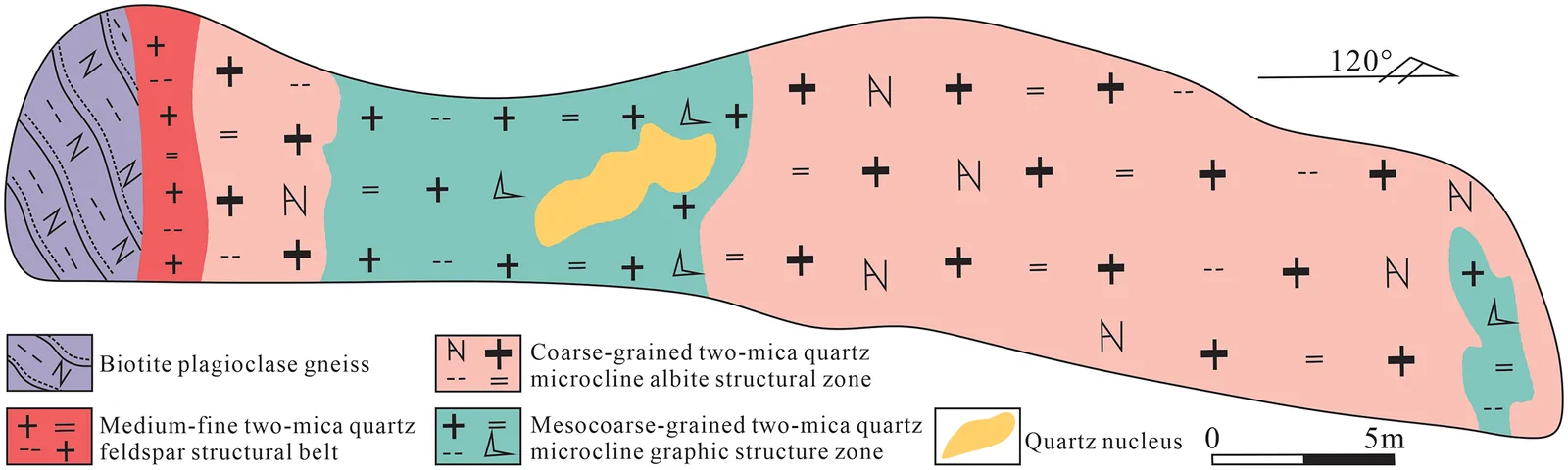
Zoning characteristics of π605 pegmatite structure.

**Fig 4:**
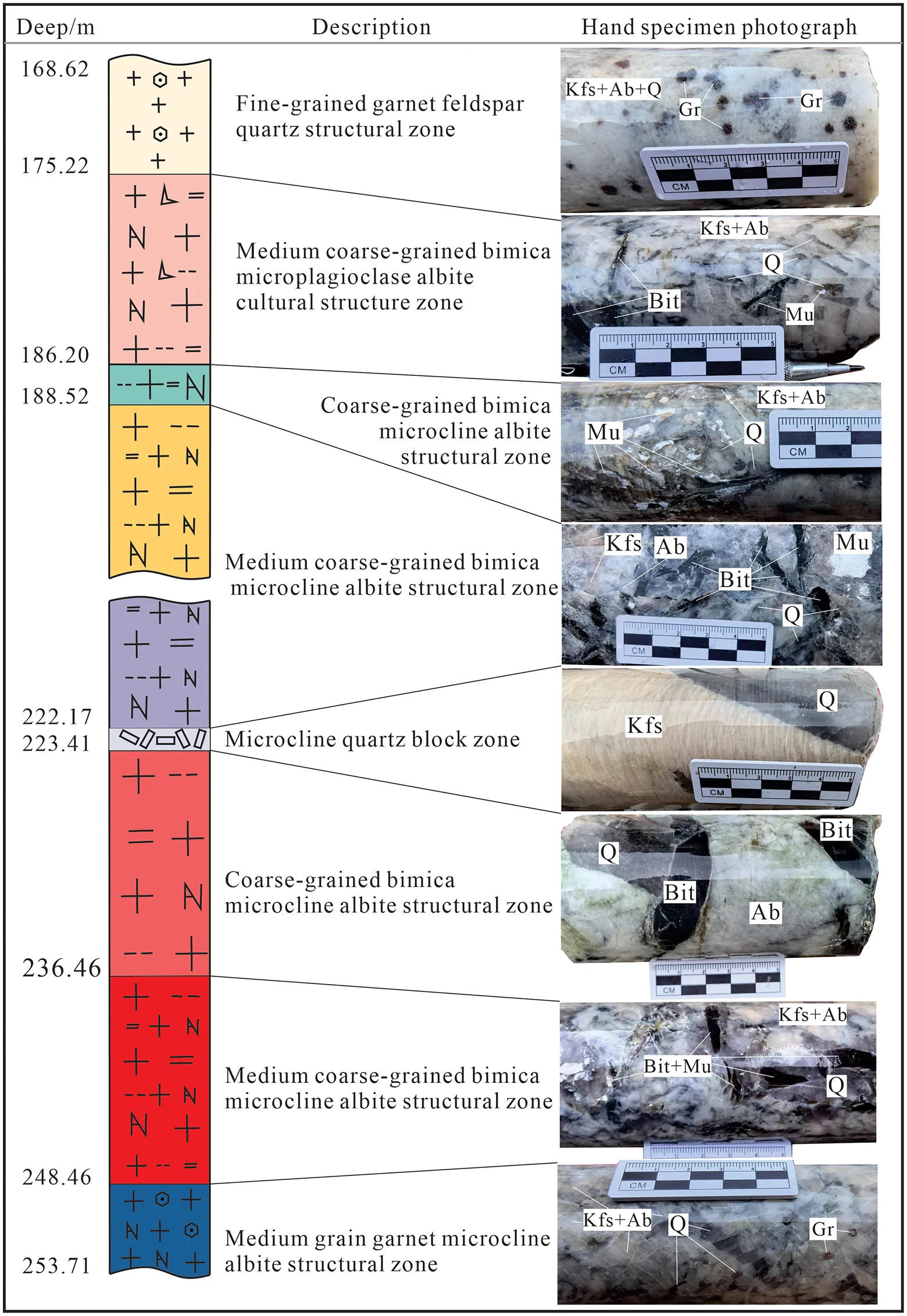
Structural zoning and rock core characteristics of HPQ pegmatite vein (π137) in ZK8–701 borehole.

High–purity quartz pegmatites exhibit a characteristic zonal structure, which is a notable difference from pegmatites associated with regional rare–metal and uranium mineralization [17]. The latter generally lack structural zoning or display only weak zonation [18]. Previous studies have summarized a typical symmetric zonation model for pegmatites in the East Qinling region, progressing from the periphery to the core as follows: granite–textured zone, pegmatitic–textured zone, graphic–textured zone, and massive–textured zone [9]. In smaller pegmatites, these structural zones are often incompletely developed, while larger pegmatites typically exhibit fully developed zones. For instance, the π605 vein in the Daxigou area shows a relatively symmetric development of medium–fine grained two–mica quartz–feldspar zones, coarse grained two–mica quartz microcline albite zones, medium–coarse grained two–mica quartz albite graphic zones, and quartz massive zones (Fig 3). Similarly, the π137 vein, as observed in drill cores, comprises eight structural zones from top to bottom: fine grained garnet–bearing quartz feldspar zone, two–mica microcline albite graphic zone, coarse grained two–mica microcline albite zone, medium–coarse grained two–mica microcline albite zone, microcline quartz massive zone, coarse grained two–mica microcline albite zone, medium–coarse grained two–mica microcline albite zone, and medium grained garnet–bearing microcline albite zone (Fig 4).

Preliminary purification of quartz from pegmatite veins, including magnetic separation, flotation, and acid leaching processes, revealed that the SiO_2_ content in both surface and drill core samples consistently exceeded 99.99%. Among the 19 samples analyzed, 9 achieved or exceeded 99.995%, with a maximum SiO_2_ content of 99.997%. Overall, quartz from drill core samples exhibited better purification outcomes compared to surface samples (Table 1). Specifically, eight structural zones of the π137 pegmatite vein, as defined in drill core samples, were subjected to initial separation and purification (samples ZK8–701–H1 to H8 correspond to the eight structural zones from top to bottom in Fig 4). Results indicated that the microcline–quartz massive zone (ZK8–701–H5) achieved the best purification results, followed by the medium–coarse grained two–mica microcline albite zone and the coarse–grained two–mica microcline albite zone. In contrast, samples from structural zones at the vein margins showed relatively poorer purification outcomes. This may be attributed to contamination from wall rock assimilation during vein emplacement, leading to higher impurity levels in quartz near the vein margins. Overall, coarser grained structural zones in the pegmatite tend to yield better quartz purification results. However, aside from the marginal zones, the differences in purification efficiency among structural zones were generally minor.

**Table 1:** Impurity content of quartz in pegmatites in Shangnan area after primary purification (wt.%).

No.	vein	samples	Al	Ca	Fe	K	Mg	Na	Ti	B	Cr	Cu	Li	Mn	Ni	Total	SiO_2_ content	Source
1	π137	GDK-6	20.80	5.86	0.94	1.78	0.44	0.70	5.75	0.32	0.00	0.02	4.32	0.45	0.12	41.45	99.9959	Surface
2	π137	TC57H1	36.40	2.85	2.96	5.42	0.26	5.58	4.74	0.29	0.21	0.08	0.84	0.06	0.00	59.69	99.9940	
3	π137	TC57H2	68.40	4.65	2.26	15.40	0.26	21.80	5.67	0.16	0.04	0.00	1.32	0.18	0.06	120.19	99.9880	
4	π137	TC57H3	47.31	3.35	1.99	6.87	0.25	7.75	4.58	0.22	0.05	0.01	0.98	0.03	0.00	73.39	99.9927	
5	π137	TC57H4	28.76	4.07	0.87	4.85	0.24	6.78	3.69	0.08	0.02	0.00	1.47	0.07	0.01	50.91	99.9949	
6	π137	ZK8–701-H1	38.63	15.30	6.48	9.62	3.25	11.99	2.80	1.48	0.11	0.20	0.97	2.28	0.00	93.11	99.9907	Drill
7	π137	ZK8–701-H2	41.92	0.00	2.33	10.48	0.00	2.66	7.69	1.33	0.00	0.23	2.25	0.18	0.00	69.07	99.9931	
8	π137	ZK8–701-H3	35.53	1.26	2.52	6.14	0.23	5.85	5.84	1.65	0.03	0.45	1.36	0.10	0.00	60.95	99.9939	
9	π137	ZK8–701-H4	42.24	5.16	11.97	7.32	0.53	6.51	4.78	1.40	0.00	0.51	1.75	2.13	0.00	84.30	99.9916	
10	π137	ZK8–701-H5	19.55	0.00	0.08	1.90	0.00	0.23	5.55	2.37	0.00	0.22	0.52	0.02	0.00	30.43	99.9970	
11	π137	ZK8–701-H6	26.15	3.70	1.19	6.37	0.00	5.18	5.26	1.45	0.00	0.07	1.41	0.05	0.00	50.84	99.9949	
12	π137	ZK8–701-H7	15.56	0.00	2.50	3.38	0.00	4.25	7.58	1.74	0.00	0.14	1.84	0.09	0.00	37.08	99.9963	
13	π137	ZK8–701-H8	31.60	8.67	2.73	13.79	0.65	8.60	6.89	1.54	0.01	0.15	1.19	0.14	0.00	75.96	99.9924	
14	π22	TC71-H1	20.36	0.00	3.60	3.96	0.47	6.14	3.17	1.78	0.32	0.30	2.93	0.44	0.00	43.46	99.9957	Surface
15	π22	TC71-H2	28.27	10.98	3.09	3.52	1.37	14.02	2.81	2.30	0.05	0.22	2.47	0.34	0.00	58.58	99.9941	
16	π22	TC71-H3	22.98	9.09	1.60	0.86	1.54	15.61	2.87	2.48	0.18	0.17	2.67	0.31	0.00	60.38	99.9940	
17	π22	ZK9–1601-H1	16.76	7.69	0.31	4.37	0.23	8.01	3.21	0.84	0.00	0.48	2.62	0.07	0.00	44.59	99.9955	Drill
18	π22	ZK9–1601-H2	14.17	4.75	0.00	5.19	0.03	9.12	3.42	0.59	0.00	0.33	2.40	0.05	0.00	40.05	99.9960	
19	π22	ZK9–1601-H3	17.05	6.82	1.93	3.88	0.14	7.26	3.34	0.01	0.00	0.20	1.21	0.24	0.10	42.18	99.9958	
20		IOTA-STD	16.20	0.50	0.23	0.60	<0.05	0.90	1.10	0.08	<0.05	<0.05	0.90	<0.05	<0.05	<19.6	>99.998	[5]

Note: The analysis results of samples No.1–19 in the table are derived from the 2024 work summary report of rare metal ore survey in Fenghuangzhai area, Shangnan County, Shaanxi Province [12]. A/CNK = molar Al_2_O_3_/(CaO + Na_2_O + K_2_O); A/NK = molar Al_2_O_3_/(Na_2_O + K_2_O); AR (Alkalinity Ratio) = (Na_2_O + K_2_O)/Al_2_O_3_; DI (Differentiation Index) = normative Qz + Or + Ab + Ne + Kp + Lc

**Fig 5:**
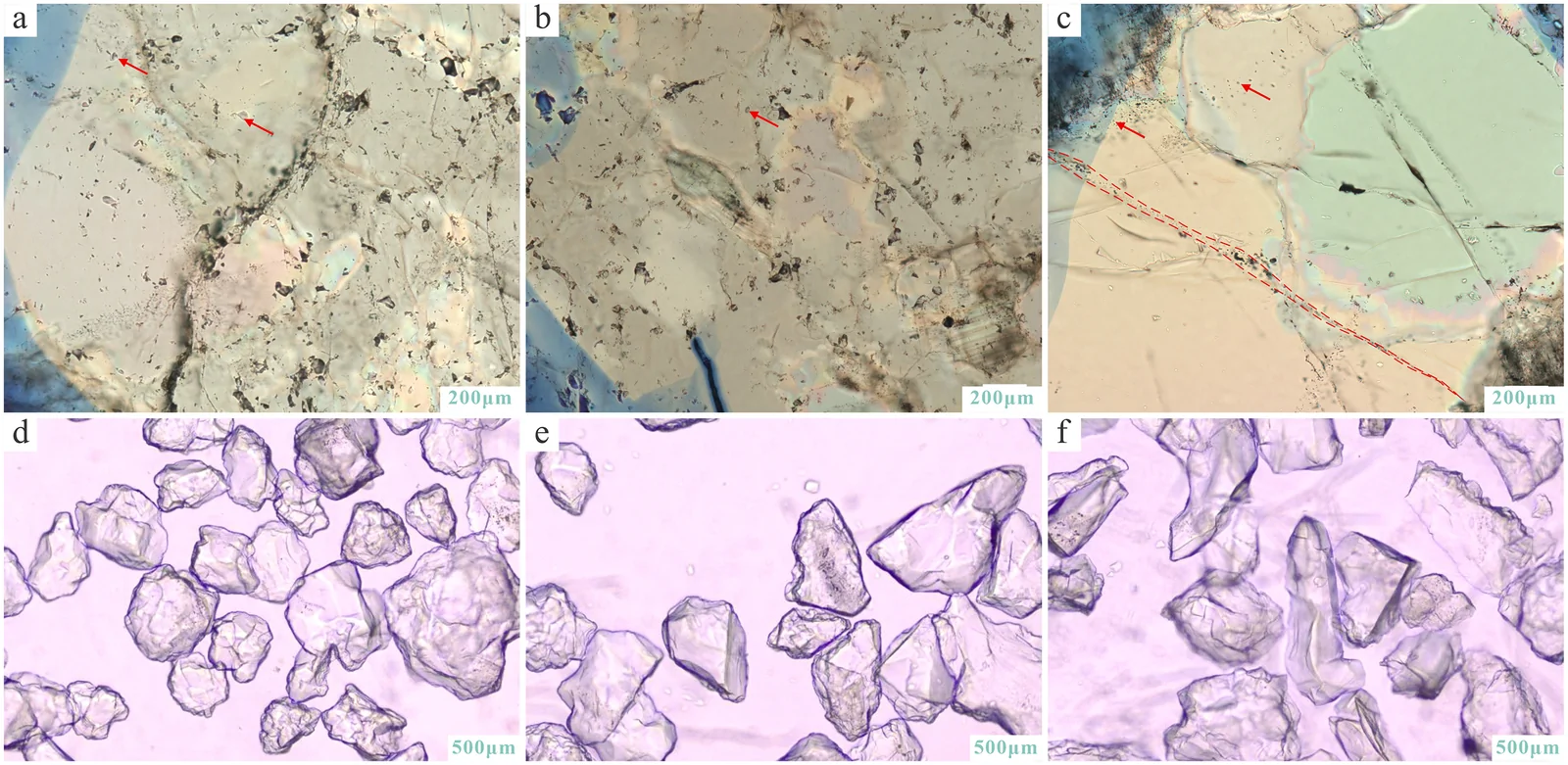
Microscopic characterization of quartz in pegmatite (π137 and π22) after primary purification. a.b–π137 pegmatite vein ore quartz fluid inclusion microscopic characteristics; the microscopic characteristics of quartz fluid inclusions in the proto–ore of c–π22 pegmatite vein; the microscopic characteristics of quartz sand inclusions in d, e–π137 pegmatite dikes (corresponding to sample ZK8-701-H5 and sample ZK8-701-H7 in Table 1, respectively); microscopic characteristics of f–π22 quartz sand inclusions (corresponding to ZK9-1601-H2 and ZK9-1601-H3 in Table 1).

As shown in Fig 5, the quartz fluid inclusions of the π137 vein ore samples are less, mostly star–shaped distribution, and more than 5μm (Figs 5a, 5b); the purified quartz sand inclusions are less and the product performs better (Figs 5d, 5e). Quartz inclusions of π22 vein ore samples are very few, and quartz mineral edges or fissures are distributed with multiple fluid inclusions, most of which are greater than 5μm (Fig 5c). Inclusions are rarely found in the purified quartz sand under the microscope (Fig 5f).

## 4. Methods

### 4.1. Field sampling and petrography

Geochemical samples for major and trace element analysis were collected from six pegmatite veins (π137, π148, π623–1, π605, π603, and π22). Each purified samples yielded a SiO_2_ purity exceeding 99.99%. To ensure representativeness and minimize compositional variability across different structural zones within the pegmatites, samples were collected from fresh outcrops using multi–point block sampling techniques. Prior to laboratory analysis, all samples were visually inspected under a petrographic microscope to exclude weathered, altered, or contaminated fractions, ensuring only fresh, unaltered mineral aggregates were selected for geochemical testing. All analyses were performed by Wuhan Shangpu Analysis Technology Co., Ltd with accreditation for geochemical testing, which holds national accreditation for geochemical testing.

### 4.2. XRF analysis

Major element analysis was conducted using a PANalytical Axios X–ray fluorescence spectroscopy (XRF) equipped with a Rh target X–ray tube (operating conditions: 40 kV, 60 mA). To ensure analytical accuracy and precision, a rigorous calibration procedure was implemented, and element-specific calibration curves for all target major elements (Si, Al, Fe, Ca, Mg, Na, K, Ti, P, Mn, etc.) were established and validated. The analytical precision and accuracy were both better than 5%, verified by repeatedly analyzing the CRMs (GBW07103, GBW07105, GBW07111) alongside the unknown samples in each analytical batch. The relative error (RE) between the measured values and certified values of CRMs was ≤ ±4% for all major elements, confirming the reliability of the XRF analysis results. For detailed procedures and data calibration methods, see reference [10].

### 4.3. ICP–MS analysis

Trace element analysis was performed using an Agilent 7900 Inductively Coupled Plasma Mass Spectrometry (ICP–MS) under optimized operating conditions. Comprehensive QA/QC measures included: (1) Calibration using multi–element standard solutions traceable to international standards. (2) The analysis of CRMs (GSR, JA-2, and JA-3) during each analytical batch to monitor instrumental drift and accuracy. (3) Precision checks via repeated measurements of selected samples showed RSDs typically < 5% for most elements. Analytical quality control data for the certified reference materials (CRMs) are summarized in Supplementary S1 File; detailed analytical procedures can be found in Reference [13].

### 4.4. Comparative Data from Previous Studies

Additionally, this study incorporates data from previous research to compare of the geochemical characteristics of pegmatites associated with different types of mineralization [19]. These include data from the Longquanping HPQ deposit in the southeastern contact zone of the Huichizi pluton, the Guangshigou–Xiaohuacha uranium deposits in the southern contact zone, and the Guanpo lithium–beryllium rare–metal pegmatites in the northwestern contact zone of the identical pluton [5,10,11,13,17,19–21]. Furthermore, data from the Ziyu rubidium pegmatites in the southern contact zone of the Kuanping pluton are included to compare pegmatite characteristics across distinct geological settings [22]. All comparative datasets were screened to ensure consistency with the analytical standards adopted in this study to avoid biases in the comparative analysis. The calibration parameters for XRF major element analysis are listed in Supplementary S2 File.

## 5. Geochemical characteristics of pegmatite

**Fig 6:**
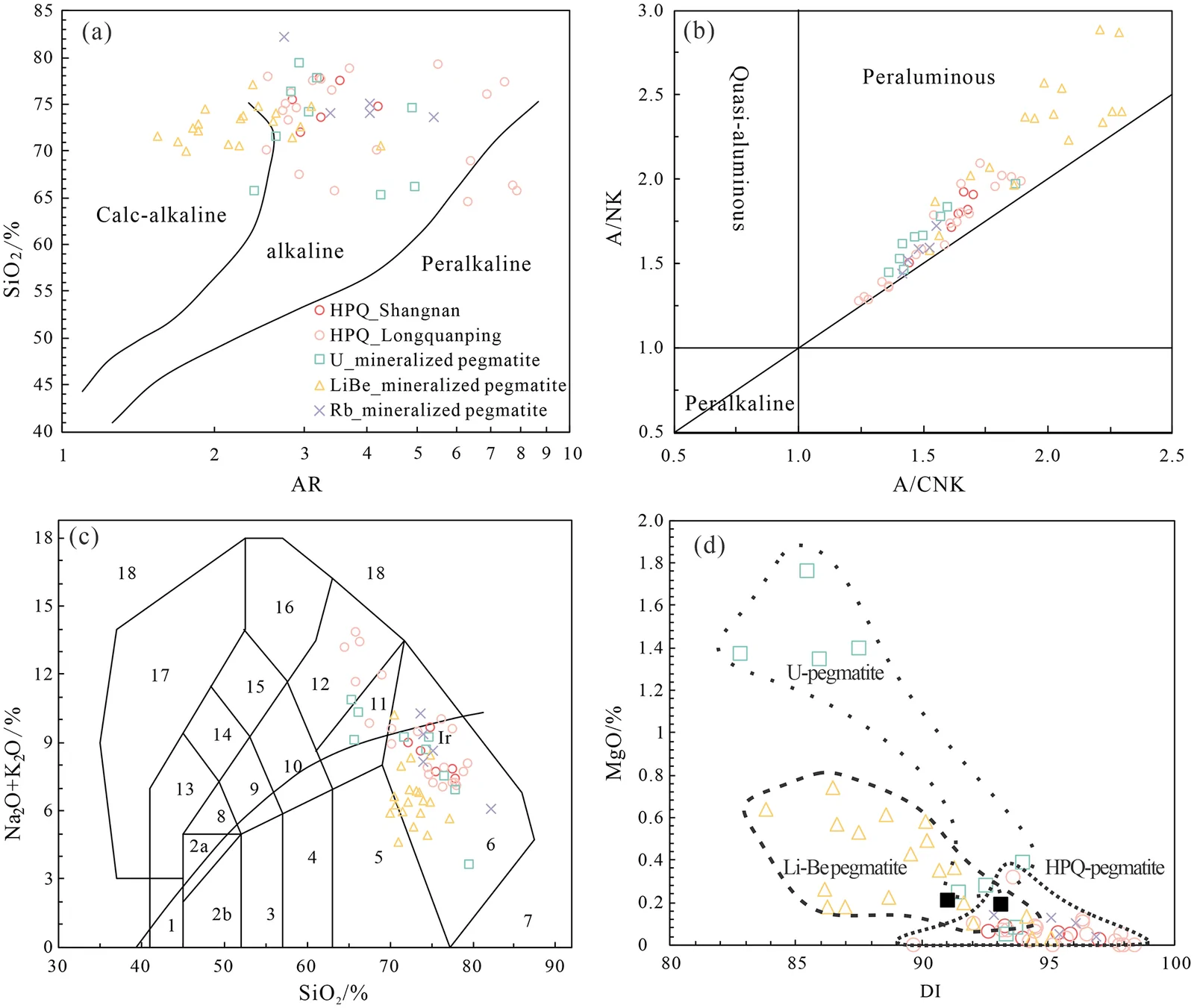
Geochemical diagram of major elements in pegmatite in the research area and its surrounding areas (adapted from [5,10,11,13,16,18,20,23]).

**Fig 7:**
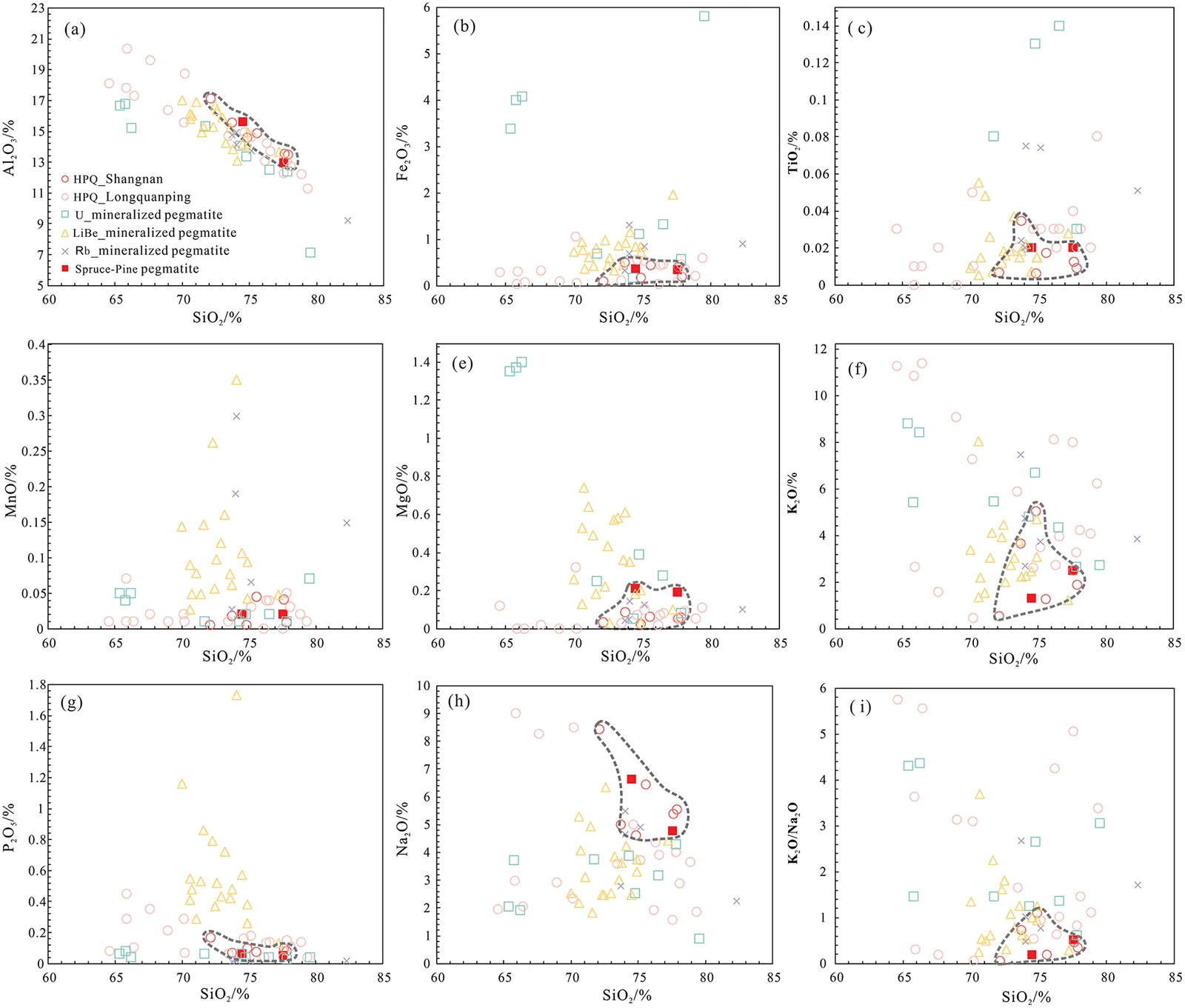
Huck diagram of major elements of pegmatite in the research area and its surrounding area.

As shown in Table 2 and Fig 6, the HPQ pegmatites in the research area are generally classified as peraluminous alkaline granites. The major element characteristics indicate that, compared to rare–metal and uranium mineralized pegmatites in the Shangnan–Lushi region, the HPQ pegmatites exhibit distinctly lower contents of Fe, Mn, Mg, P, and Ti, as well as higher Na contents and a significantly lower K/Na ratio. Fig 7 shows that the data points for HPQ pegmatites from the Shangnan region cluster within a relatively narrow range, similar to those of the Spruce–Pine HPQ pegmatites. Compared to the Longquanping HPQ pegmatites, the major element composition of the Shangnan pegmatites more closely resembles that of the Spruce–Pine pegmatites.

**Table 2:** Content table of major elements of pegmatite in the research area and surrounding areas (wt.%).

No.	Samples	Lithology	Location	type	SiO_2_	TiO_2_	Al_2_O_3_	Fe_2_O_3_	MnO	MgO	CaO	Na_2_O	K_2_O	P_2_O_5_	LOI	Total	Na_2_O+K_2_O	K_2_O/Na_2_O	A/CNK	A/NK	AR	DI	Source
1	DH1	Two–mica monzonitic pegmatite	Shangnan	HPQ	73.76	0.03	15.55	0.49	0.017	0.09	0.84	5.00	3.65	0.07	0.68	100.16	8.6474	0.73	1.64	1.80	3.24	93.33	Our team
2	DH2			HPQ	75.61	0.02	14.89	0.45	0.045	0.06	1.25	6.45	1.28	0.07	0.42	100.53	7.7205	0.20	1.66	1.93	2.83	92.63	
3	DH3			HPQ	72.16	0.01	17.12	0.10	0.005	0.03	1.09	8.44	0.53	0.17	0.48	100.13	8.9705	0.06	1.70	1.91	2.94	94.02	
4	DH4			HPQ	74.89	0.01	14.56	0.17	0.005	0.02	0.43	4.61	5.04	0.10	0.40	100.23	9.6511	1.09	1.44	1.51	4.20	97.06	
5	DH5			HPQ	77.88	0.01	13.47	0.19	0.009	0.05	0.64	5.53	1.87	0.08	0.48	100.21	7.3982	0.34	1.68	1.82	3.21	95.43	
6	DH6			HPQ	77.65	0.01	13.52	0.39	0.041	0.05	0.55	5.38	2.47	0.10	0.44	100.61	7.8568	0.46	1.61	1.72	3.53	95.90	
7	D01	Muscovite monzpegmatite	Longquanping	HPQ	65.90	0.01	20.35	0.30	0.070	0.00	0.80	8.99	2.65	0.45	0.63	100.14	11.64	0.29	1.64	1.75	3.45	95.209	[11]
8	D02			HPQ	67.63	0.02	19.56	0.33	0.020	0.02	0.50	8.25	1.58	0.35	1.01	99.27	9.83	0.19	1.89	1.99	2.92	94.269	
9	D04			HPQ	65.86	0.00	17.78	0.04	0.010	0.00	0.07	2.99	10.84	0.29	0.26	98.15	13.83	3.63	1.28	1.29	7.88	98.429	
10	D08			HPQ	68.99	0.00	16.33	0.09	0.010	0.00	0.06	2.90	9.06	0.21	0.42	98.06	11.96	3.12	1.36	1.37	6.40	97.827	
11	D11	Two–mica monzpegmatite		HPQ	70.18	0.05	15.54	1.06	0.020	0.32	0.18	2.35	7.27	0.29	0.51	97.75	9.62	3.09	1.59	1.62	4.15	93.622	
12	D14	Muscovite albite pegmatite		HPQ	74.66	0.03	14.90	0.55	0.020	0.07	0.75	4.98	2.62	0.16	0.98	99.71	7.6	0.53	1.78	1.96	2.89	93.202	
13	D15			HPQ	77.84	0.02	13.07	0.45	0.050	0.06	0.67	3.99	3.25	0.15	0.80	100.36	7.24	0.81	1.65	1.81	3.23	94.487	
14	D19	Muscovite monzpegmatite		HPQ	76.33	0.02	14.23	0.43	0.040	0.07	0.62	4.36	2.71	0.13	0.89	99.82	7.07	0.62	1.85	2.01	2.82	93.429	
15	D22	Muscovite albite pegmatite		HPQ	75.19	0.03	14.62	0.54	0.030	0.10	0.84	3.72	3.51	0.18	1.09	99.85	7.23	0.94	1.81	2.02	2.76	92.039	
16	D24	Two–mica monzpegmatite		HPQ	66.42	0.01	17.30	0.07	0.010	0.00	0.12	2.05	11.38	0.10	0.29	97.75	13.43	5.55	1.28	1.29	7.73	98.031	
17	D25	Tourmaline–bearing Muscovite monzpegmatit		HPQ	73.48	0.02	14.68	0.11	0.010	0.03	0.52	3.58	5.88	0.06	0.44	98.81	9.46	1.64	1.47	1.55	2.78	95.764	
18	D27			HPQ	64.61	0.03	18.11	0.28	0.010	0.12	0.08	1.96	11.25	0.08	0.42	96.95	13.21	5.74	1.36	1.37	6.31	96.386	
19	D29			HPQ	70.24	0.01	18.73	0.06	0.010	0.00	1.93	8.48	0.45	0.07	0.31	100.30	8.93	0.05	1.72	2.10	2.52	89.688	
20	LD05-T1	Two–mica Microcline pegmatite	Longquanping	HPQ	78.13	0.03	12.74	0.36	0.030	0.09	0.49	2.87	4.21	0.11	/	99.05	7.08	1.47	1.68	1.80	2.53	94.501	[13]
21	LD05-T2			HPQ	78.89	0.02	12.22	0.21	0.020	0.05	0.48	3.64	4.07	0.14	/	99.74	7.71	1.12	1.49	1.58	3.69	96.536	
22	LD05-T3			HPQ	76.56	0.03	13.70	0.45	0.040	0.08	0.64	3.91	3.97	0.14	/	99.52	7.88	1.02	1.61	1.74	3.40	94.529	
23	LD10-T1			HPQ	79.37	0.08	11.30	0.59	0.010	0.11	0.39	1.85	6.24	0.04	/	99.98	8.09	3.37	1.33	1.40	5.49	96.389	
24	LD10-T2			HPQ	76.18	0.03	13.08	0.03	0.000	0.02	0.36	1.91	8.11	0.04	/	99.77	10.02	4.25	1.26	1.31	6.86	97.668	
25	LD10-T3			HPQ	77.55	0.04	12.24	0.09	0.000	0.05	0.30	1.58	7.99	0.04	/	99.88	9.57	5.06	1.24	1.28	7.44	97.882	
27	SP01*	High–purity quartz Mineralized pegmatite	Spruce–Pine	HPQ	74.50	0.02	15.60	0.37	0.020	0.21	1.54	6.62	1.29	0.06	/	100.23	7.91	0.19	1.65	1.97	2.71	91.045	[20]
28	SP02*			HPQ	77.60	0.02	12.95	0.34	0.020	0.19	1.15	4.76	2.49	0.05	/	99.57	7.25	0.52	1.54	1.79	3.12	93.17	
29	PD9851	The edge zone of biotite pegmatite	Guangshigou	U	66.23	0.64	15.2	4.07	0.050	1.4	0.38	1.93	8.41	0.04	1.6	99.95	10.34	4.36	1.42	1.47	4.95	87.505	[16]
30	PD10353			U	65.38	0.51	16.63	3.39	0.050	1.35	0.96	2.05	8.82	0.06	0.71	99.9	10.87	4.30	1.41	1.53	4.24	85.955	
31	PD10804			U	74.79	0.13	13.35	1.11	0.020	0.39	0.59	2.52	6.7	0.04	0.32	99.91	9.22	2.66	1.36	1.45	4.91	94.013	
32	PD9454	The inner zone of biotite pegmatite		U	76.54	0.14	12.51	1.32	0.020	0.28	0.86	3.18	4.33	0.04	0.77	99.98	7.51	1.36	1.49	1.67	2.81	92.543	
33	PD98511			U	71.72	0.08	15.3	0.69	0.010	0.25	1.24	3.73	5.47	0.06	1.35	99.9	9.2	1.47	1.47	1.66	2.64	91.441	
34	3GSG40			U	74.34	0.02	14.05	0.16	0.010	0.05	1.23	3.87	4.82	0.06	0.56	99.16	8.69	1.25	1.42	1.62	3.05	93.373	
35	XHC-14	biotite pegmatite	Xiaohuacha	U	79.56	0.84	7.13	5.81	0.070	1.76	0.21	0.89	2.72	0.04	1.03	100.2	3.61	3.06	1.87	1.98	2.94	85.447	[19]
36	XHC-6(2)			U	77.86	0.03	12.38	0.57	0.010	0.08	0.95	4.28	2.66	0.03	1.09	99.96	6.94	0.62	1.57	1.78	3.17	93.728	
37	PD945−9			U	65.81	0.53	16.77	3.99	0.040	1.37	1.4	3.72	5.40	0.08	0.99	100.17	9.12	1.45	1.59	1.84	2.39	82.814	
38	HQ1	biotite granitic pegmatite	Guanpo	Li/Be	74.13	0.02	13.09	1.16	0.350	0.35	1.29	4.21	2.25	1.73	/	/	6.46	0.53	1.69	2.03	2.63	90.717	[10]
39	HQ2			Li/Be	71.46	0.03	14.93	0.42	0.048	0.49	1.68	4.94	3.03	0.53	/	/	7.97	0.61	1.55	1.87	2.84	90.208	
40	HQ3			Li/Be	71.11	0.05	16.87	0.62	0.078	0.64	1.11	3.09	1.52	0.29	/	/	4.61	0.49	2.95	3.66	1.69	83.811	
41	HQ4	Spodumene–lepidolite pegmatite		Li/Be	73.62	0.02	15.19	0.43	0.077	0.36	0.51	3.02	3.77	0.42	/	/	6.79	1.25	2.08	2.24	2.25	91.283	
42	HQ5			Li/Be	72.47	0.02	16.64	0.43	0.056	0.43	0.46	2.46	4.46	0.37	/	/	6.92	1.81	2.25	2.40	1.81	89.6	
43	HQ6			Li/Be	72.98	0.02	15.92	0.32	0.120	0.57	0.94	2.53	2.72	0.43	/	/	5.25	1.08	2.57	3.03	1.86	86.674	
44	HQ7	Rare metal mineralized pegmatite		Li/Be	70.78	0.02	15.99	0.37	0.049	0.74	1.50	4.08	2.20	0.48	/	/	6.28	0.54	2.06	2.55	2.12	86.474	
45	HQ8			Li/Be	73.79	0.02	13.84	0.92	0.061	0.61	1.25	3.62	2.24	0.48	/	/	5.86	0.62	1.95	2.36	2.27	88.608	
46	HQ9			Li/Be	70.62	0.06	15.76	0.93	0.089	0.53	1.63	5.29	1.35	0.55	/	/	6.64	0.26	1.91	2.37	2.24	87.491	
47	HQ10			Li/Be	73.26	0.04	14.23	0.86	0.160	0.58	1.21	3.83	3.03	0.72	/	/	6.86	0.79	1.76	2.07	2.60	90.149	
48	B1	Spodumene–lepidolite pegmatite	Guanpo	Li/Be	72.32	0.012	15.26	0.97	0.261	0.22	1.17	2.45	3.93	0.79	2.55	99.93	6.38	1.60	2.02	2.39	1.85	88.701	[23]
49	B2			Li/Be	70.03	0.009	16.97	0.73	0.143	0.26	1.53	2.52	3.38	1.16	3.15	99.9	5.9	1.34	2.28	2.88	1.75	86.155	
50	B3			Li/Be	71.64	0.007	15.26	0.8	0.146	0.18	1.78	1.82	4.1	0.86	3.29	99.9	5.92	2.25	1.98	2.58	1.54	86.275	
51	B4			Li/Be	74.51	0.007	14.19	0.84	0.106	0.18	1.51	2.45	2.46	0.57	3.03	99.87	4.91	1.00	2.21	2.89	1.91	86.98	
52	B5	Tourmaline–muscovite pegmatite		Li/Be	77.24	0.028	13.62	1.96	0.047	0.1	0.27	4.43	1.24	0.13	0.84	99.9	5.67	0.28	2.29	2.40	2.38	92.04	
53	B6			Li/Be	72.64	0.016	16.35	0.59	0.097	0.03	0.47	6.33	1.98	0.52	0.9	99.93	8.31	0.31	1.86	1.97	2.95	94.384	
54	B7			Li/Be	74.9	0.015	14.05	0.72	0.093	0.03	0.56	3.74	4.69	0.38	0.71	99.89	8.43	1.25	1.56	1.67	3.10	95.169	
55	B8			Li/Be	74.91	0.015	14.93	0.85	0.042	0.2	0.34	3.31	3.08	0.26	2.01	99.92	6.39	0.93	2.22	2.34	2.44	91.673	
56	B9			Li/Be	70.65	0.005	16.13	0.76	0.027	0.13	0.37	2.18	8.03	0.41	1.2	99.9	10.21	3.68	1.52	1.58	4.25	94.175	
57	2	Microplagioclase granite pegmatite	Ziyu	Rb	74.08	0.075	14.09	1.3	0.298	0.142	0.918	5.49	2.67	0.016	0.24	99.32	8.16	0.49	1.55	1.73	3.38	92.883	[21]
58	3			Rb	74.07	0.02	14.93	0.682	0.190	0.047	0.435	4.64	4.71	0.02	0.22	99.96	9.35	1.02	1.53	1.60	4.05	95.45	
59	4			Rb	73.75	0.024	14.76	0.322	0.027	0.04	0.154	2.79	7.47	0.012	0.17	99.52	10.26	2.68	1.42	1.44	5.41	96.959	
60	5			Rb	82.33	0.051	9.22	0.899	0.148	0.098	0.356	2.23	3.83	0.017	0.47	99.65	6.06	1.72	1.44	1.52	2.74	96.132	
61	6			Rb	75.18	0.074	13.69	0.844	0.065	0.124	0.619	4.9	3.74	0.012	0.53	99.78	8.64	0.76	1.48	1.58	4.05	95.165	

Note: A/CNK = molar Al_2_O_3_/(CaO + Na_2_O + K_2_O); A/NK = molar Al_2_O_3_/(Na_2_O + K_2_O); AR (Alkalinity Ratio) = (Na_2_O + K_2_O)/Al_2_O_3_; DI (Differentiation Index) = normative Qz + Or + Ab + Ne + Kp + Lc.

**Fig 8:**
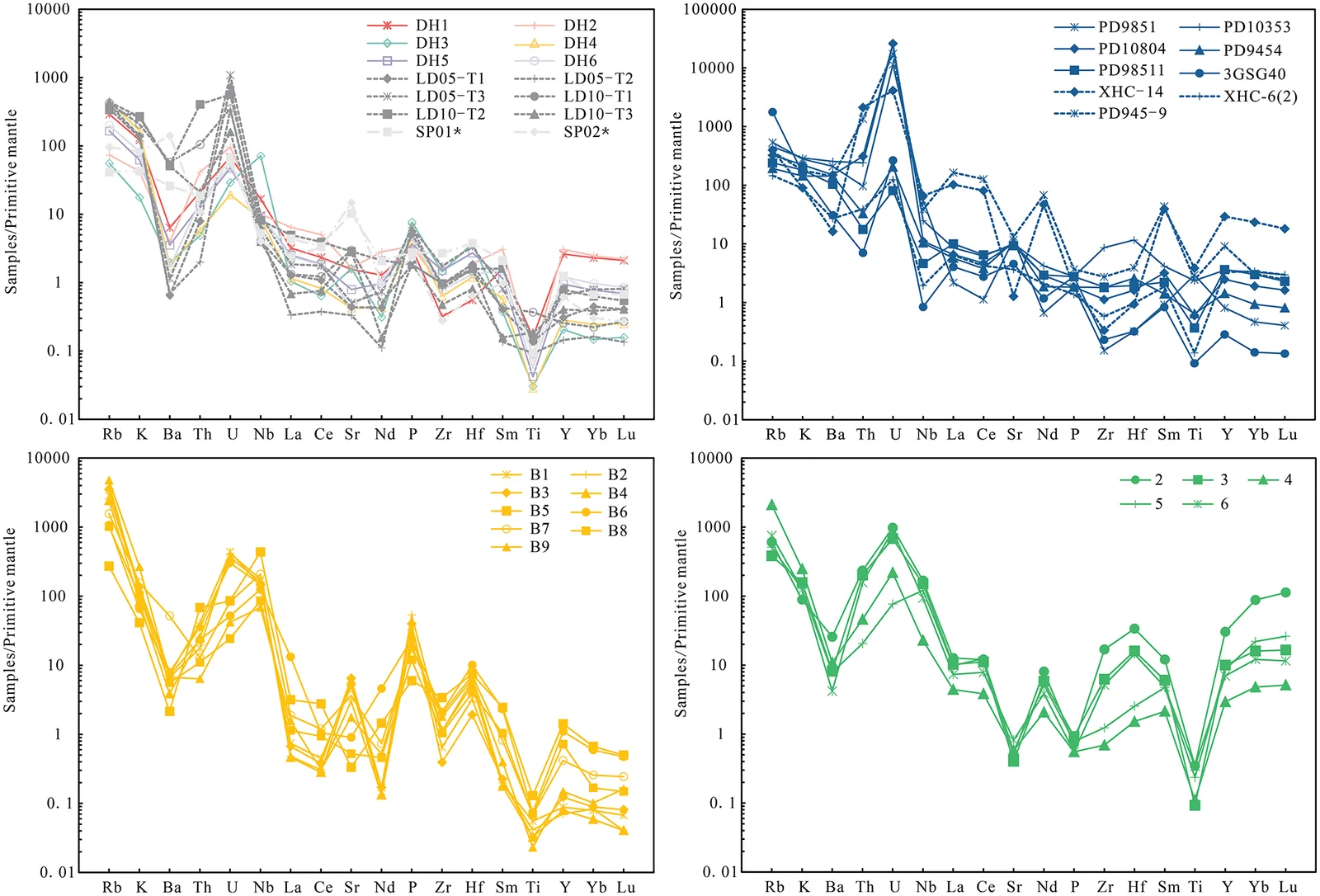
Primitive mantle–normalized trace element spider diagram of pegmatite in the research area and surrounding areas. a, b, c and d are Shangnan HPQ pegmatite, Shangnan uranium mineralized pegmatite,Guanpo rare metal mineralized pegmatite and Ziyu rubidium mineralized pegmatite, respectively. SP01 * and SP02 * are Spruce–Pine HPQ pegmatite samples from [20]).

The primitive mantle–normalized trace element spider diagrams of various types of pegmatites have certain similarities. HPQ pegmatites are enriched in U, P, and Hf, with particularly enrichment of uranium. These pegmatites show strong depletion in Ba and Ti and moderate depletion in Nd and Zr (Fig 8), indicating that it has undergone a high degree of crystalline differentiation and intense melt-fluid interaction. Among the different types of pegmatites, those with lithium–beryllium mineralization share the closest trace element characteristics with HPQ pegmatites. However, lithium–beryllium mineralized pegmatites exhibit significant enrichment in Rb and Nb, which is absent in HPQ pegmatites. In contrast, the trace element characteristics of HPQ pegmatites differ markedly from those of uranium–mineralized pegmatites in Guangshigou and Xiaohuacha, Shangnan (Fig 8b), and rubidium–mineralized pegmatites in Ziyu (Fig 8d). The latter two types exhibit strong depletion of Sr and P in some or all samples. The rubidium–mineralized pegmatites in Ziyu show the most pronounced differences, characterized by significant enrichment in Y, Yb, and Lu [22].

**Fig 9:**
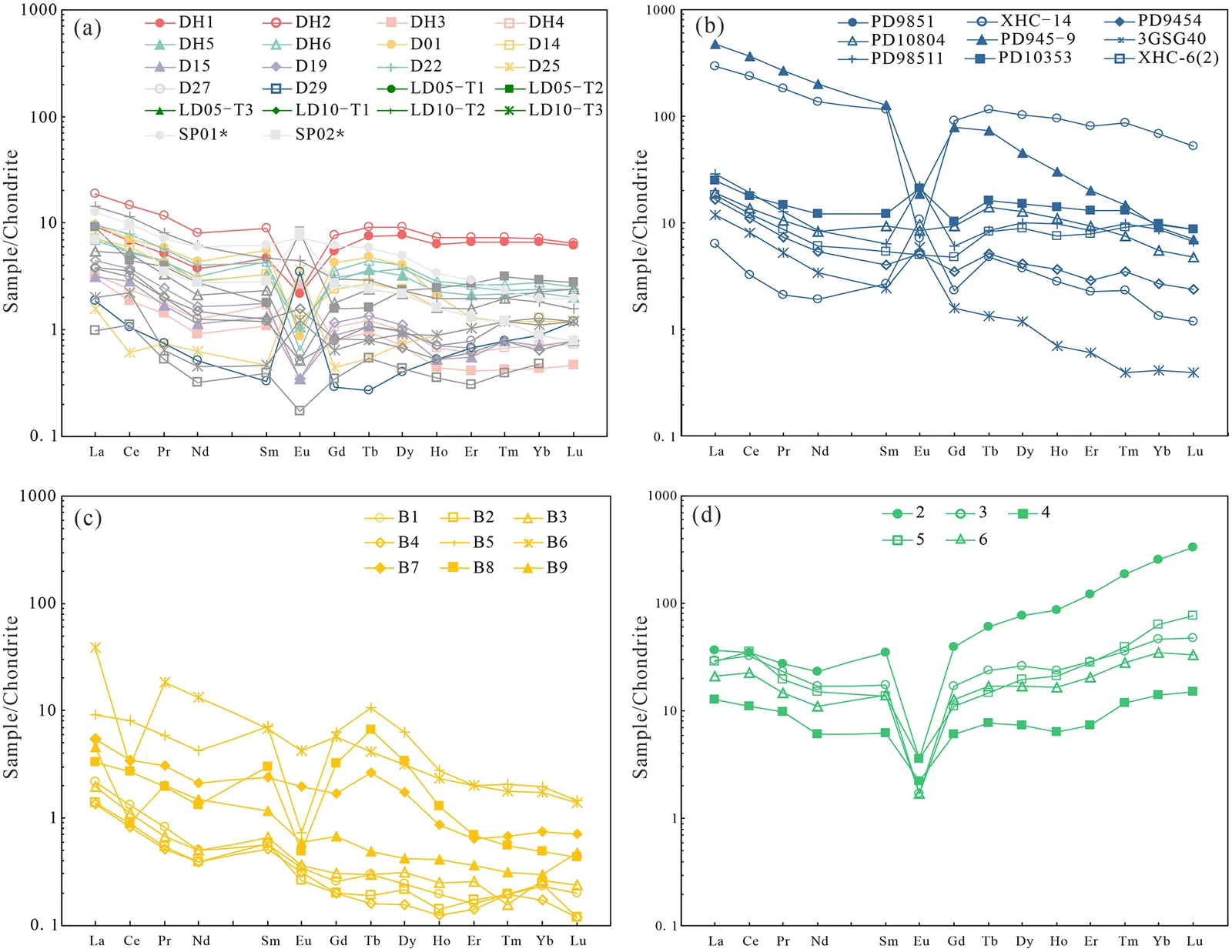
The standardized distribution pattern of rare earth elements of pegmatite in the study area and its surrounding areas. a, b, c and d are Shangnan HPQ pegmatite, Shangnan uranium mineralized pegmatite, Lushiguanpo rare metal mineralized pegmatite and Ziyu rubidium mineralized pegmatite, respectively. SP01 * and SP02 * are Spruce–Pine HPQ pegmatite samples in Fig a (according to [20].

The total rare earth element (REE) content of HPQ pegmatites in the Shangnan ranges from 3.28 × 10^−6^ to 15.66 × 10^−6^, indicating relatively low levels overall. The contents of light rare earth elements (LREE) and heavy rare earth elements (HREE) show minimal differences, with LREE/HREE values ranging from 1.51 to 4.82 and La_N_/Yb_N_ values from 1.39 to 7.12, suggesting weak differentiation between LREE and HREE. The chondrite–normalized REE patterns exhibit a slightly right–tilted flat trend, with most samples displaying a pronounced negative Eu anomaly and a weak negative Ce anomaly. The Eu anomaly (δ_Eu_) ranges from −0.25 to −0.43 (except for sample DH3, which shows a positive Eu anomaly with δ_Eu_ of 2.79), while the Ce anomaly (δ_Ce_) ranges from −0.83 to −0.96 (Table 3, Fig 9). A comparison with pegmatites from surrounding areas reveals that the REE characteristics of Shangnan HPQ pegmatites are remarkably similar to those of the Longquanping HPQ pegmatites and the Spruce–Pine pegmatites in terms of total REE content, LREE/HREE differentiation, and the presence of Eu anomalies (Fig 9a). In contrast, most samples of rare–metal pegmatites from Guanpo exhibit only weak negative Eu anomalies (Fig 9c). Distinct differences are observed in the Guangshigou–Xiaohuacha uranium–mineralized pegmatites and the Ziyu rubidium–mineralized pegmatites, which show significantly higher total REE contents. Particularly, rubidium–mineralized pegmatites are enriched in HREE, with chondrite–normalized REE patterns displaying a left–tilted trend (Figs 9b, 9d).

**Table 3:** The contents of trace elements and rare earth elements in pegmatites in the research area and its surrounding areas (ppm).

No.	Samples			Lithology			Location	type		Rb	Sr	Ba	Th	U	Nb	Ta	Zr	Hf	Y	La	Ce	Pr
1	DH1			Two–mica pegmatite			Shangnan	HPQ		184.52	32.84	44.39	1.81	1.44	11.95	0.88	3.55	0.17	11.86	2.19	4.15	0.49
2	DH2							HPQ		46.76	34.75	27.51	3.51	2.02	7.13	0.97	9.63	0.58	13.95	4.40	8.91	1.11
3	DH3							HPQ		35.25	33.73	13.74	0.41	0.61	50.93	9.46	16.15	1.15	0.94	0.72	1.13	0.13
4	DH4							HPQ		282.10	8.91	12.46	0.49	0.41	6.05	0.73	7.05	0.37	1.28	0.79	1.46	0.17
5	DH5							HPQ		104.69	16.65	24.73	1.14	0.99	6.70	0.68	16.80	0.84	4.29	1.73	3.29	0.42
6	DH6							HPQ		127.56	8.89	11.68	1.31	1.07	6.73	0.58	8.69	0.41	5.51	1.65	3.20	0.42
7	D01			Muscovite monzpegmatite			Longquanping	HPQ		0.9	6.58	9.39	28.84	2.83	2.34	7.26	37.02	21.43	/	2.25	4.31	0.56
8	D14			Muscovite albite pegmatite				HPQ		3.8	0.88	10.22	0.95	3.8	15.64	11.05	2.13	9.56	/	1.68	3.63	0.39
9	D15							HPQ		2.74	0.31	8.86	0.3	2.74	8.54	3.87	4.02	11.74	/	0.74	1.74	0.16
10	D19			Muscovite monzpegmatite				HPQ		0.55	3.35	6.54	12.34	4.61	0.55	5.52	3.53	29.22	/	1.05	2.26	0.23
11	D24			Two–mica monzpegmatite				HPQ		0.38	0.02	0.72	0.01	0.38	92.38	1298.38	0.02	3.69	/	0.35	0.13	0.06
12	D25			Tourmaline–bearing Muscovite monzpegmatit				HPQ		1.13	0.13	2.55	0.09	1.13	42.08	416.98	0.16	8.52	/	0.37	0.37	0.07
13	D27			Tourmaline–bearing muscovite albite pegmatite				HPQ		0.79	0.14	1.65	0.03	0.79	31.33	124.48	0.06	9.61	/	0.44	0.64	0.07
14	D29							HPQ		0.17	0.78	0.27	0.1	0.17	79.59	21.12	0.3	1.3	/	2.16	2.72	0.37
15	LD05-T1			Two–mica microcline pegmatite			Longquanping	HPQ		255	9.22	4.54	0.67	15.2	4.37	0.95	10.6	0.57	1.4	0.89	1.92	0.19
16	LD05-T2							HPQ		223	6.99	4.85	0.17	7.16	3.67	0.87	9.2	0.49	0.66	0.23	0.67	0.05
17	LD05-T3							HPQ		223	10.7	8.01	1	22.7	6.87	1.69	19.5	1.04	3	1.27	3.14	0.31
18	LD10-T1							HPQ		270	62.6	403	8.93	7.38	2.88	1.08	10.8	0.46	1.16	0.91	2.17	0.19
19	LD10-T2							HPQ		213	60.3	370	34.5	11.8	5.79	2.09	10.9	0.49	3.59	3.41	6.97	0.76
20	LD10-T3							HPQ		279	59.3	355	1.79	3.36	2.84	1.01	5.33	0.25	1.84	0.47	1.34	0.06
21	SP01*			High–purity quartz mineralized pegmatite			Spruce–Pine	HPQ		26.3	219	181	1.58	1.25	3.75	0.28	30	1.17	5.68	3	6	0.68
22	SP02*							HPQ		60.7	313	981	0.92	1.48	2.86	0.31	3.1	0.2	2.8	1.66	5.77	0.33
23	PD9851			The edge zone of biotite pegmatite			Guangshigou	U		341	246	1464	8.2	240	39.1	3.1	1.7	0.1	3.7	1.5	2	0.2
24	PD10353							U		286	212	1786	20.4	530	17.7	1.9	96.9	3.6	16.1	5.9	11	1.4
25	PD10804							U		206	215	1051	26.4	551	7.9	2	12.6	0.5	11.3	4.5	8.3	1
26	PD9454			The inner zone of biotite pegmatite				U		122	231	998	2.8	4.3	7.4	0.8	20.2	0.8	6.5	3.9	6.7	0.7
27	PD98511							U		153	197	728	1.5	1.7	3.3	0.3	20.5	0.6	16.6	6.9	11.6	1.2
28	3GSG40							U		1129	96	216	0.6	5.6	0.6	0.3	2.6	0.1	1.3	2.8	4.9	0.5
29	XHC-14			Biotite pegmatite			xiaohuacha	U		252	26.9	114	180	86.7	47.3	2.91	3.76	0.29	133	70.5	144	17.4
30	XHC-6(2)							U		92.3	77.4	189	3.35	2.61	1.39	0.2	6.5	0.3	14.9	4.38	7.48	0.82
31	PD945−9							U		238	278	993	115	365	27.4	2.32	30.8	1.21	41.7	113	225	25.4
32	B1			Spodumene–lepidolite pegmatite			Guanpo	Li/Be		1804	106	47	1.5	9.12	103	88.3	7.3	1	0.4	0.510	0.800	0.077
33	B2							Li/Be		2731	64	51	3.47	8.7	126	134.5	12.4	1.86	0.32	0.330	0.540	0.051
34	B3							Li/Be		2223	136	55	3.04	6.34	106	104.2	4.4	0.59	0.56	0.460	0.670	0.064
35	B4							Li/Be		1531	125	27	2.14	7.62	116	119.5	12.5	1.62	0.36	0.320	0.500	0.048
36	B5			Tourmaline–muscovite pegmatite				Li/Be		173	7	15	5.77	1.8	310	39.9	37.9	2.31	6.46	2.180	4.900	0.560
37	B6							Li/Be		674	19	50	1.98	1.09	89	112	23.5	3.1	5	9.060	1.860	1.752
38	B7							Li/Be		991	77	362	1.09	1.74	148	128.8	22.4	2.18	1.9	1.290	2.120	0.292
39	B8							Li/Be		645	11	39	0.94	0.51	61	32.5	11.7	1.24	3.26	0.780	1.660	0.186
40	B9							Li/Be		3028	37	47	0.54	0.88	49	40.1	20.1	1.96	0.68	1.080	0.550	0.192
41	2			Microcline granite pegmatite			Ziyu	Rb		381	10.6	179	19.9	20.5	119	8.83	188	10.4	138	8.66	21.4	2.58
42	3							Rb		242	8.5	56.9	17.1	14.3	107	6.93	69.8	4.94	45.3	7.05	19.7	2.2
43	4							Rb		1338	12.2	76	3.91	4.6	16.3	5.26	7.74	0.47	13.4	3.04	6.81	0.94
44	5							Rb		323	11.8	54.7	1.74	1.61	86.3	7.3	13.8	0.78	39.5	6.77	22	1.85
45	6							Rb		476	17.3	29.2	13.4	18.6	66.5	18.3	57.7	4.44	31.6	5.04	13.9	1.41
No.	Samples	Nd	Sm	Eu	Gd	Tb	Dy	Ho	Er	Tm	Yb	Lu	ΣREE		LREE	HREE	LREE/HREE		La_N_/Yb_N_	δEu	δCe	Source
1	DH1	1.74	0.71	0.13	1.11	0.28	1.93	0.36	1.10	0.17	1.13	0.16	15.66		9.41	6.24	1.51		1.39	0.43	0.94	Our team
2	DH2	3.81	1.36	0.13	1.56	0.34	2.30	0.42	1.20	0.19	1.22	0.16	27.11		19.72	7.39	2.67		2.59	0.28	0.96	
3	DH3	0.42	0.17	0.15	0.16	0.04	0.18	0.02	0.07	0.01	0.07	0.01	3.28		2.72	0.56	4.82		7.12	2.79	0.83	
4	DH4	0.58	0.26	0.02	0.21	0.05	0.25	0.04	0.10	0.02	0.12	0.02	4.07		3.27	0.8	4.07		4.71	0.25	0.93	
5	DH5	1.32	0.51	0.06	0.59	0.14	0.81	0.13	0.35	0.05	0.38	0.05	9.84		7.33	2.5	2.93		3.27	0.34	0.91	
6	DH6	1.47	0.66	0.04	0.73	0.17	1.03	0.16	0.44	0.07	0.48	0.06	10.57		7.44	3.13	2.38		2.5	0.16	0.92	
7	D01	2.02	0.86	0.05	0.87	0.18	1.01	0.12	0.21	0.03	0.15	0.02	12.64		10.05	2.59	3.88		10.76	0.17	0.92	[11]
8	D14	1.37	0.5	0.07	0.49	0.1	0.61	0.09	0.22	0.03	0.22	0.03	9.43		7.64	1.79	4.27		5.48	0.43	1.06	
9	D15	0.53	0.2	0.02	0.18	0.04	0.24	0.03	0.09	0.02	0.12	0.02	4.13		3.39	0.74	4.58		4.42	0.32	1.18	
10	D19	0.76	0.27	0.02	0.24	0.05	0.28	0.04	0.11	0.02	0.15	0.02	5.5		4.59	0.91	5.04		5.02	0.24	1.08	
11	D24	0.23	0.07	0.86	0.07	0.01	0.06	0.01	0.03	0.01	0.03	0	12.99		10.19	2.8	3.64		3.99	0.28	1.02	
12	D25	0.29	0.07	0.21	0.09	0.02	0.19	0.05	0.17	0.03	0.2	0.03	2.16		1.38	0.78	1.77		1.33	8.09	0.53	
13	D27	0.24	0.05	0.2	0.06	0.01	0.1	0.03	0.11	0.02	0.15	0.03	2.15		1.64	0.51	3.22		2.1	11.15	0.81	
14	D29	1.28	0.27	0.48	0.32	0.06	0.59	0.14	0.45	0.08	0.49	0.07	9.48		7.28	2.2	3.31		3.16	4.98	0.68	
15	LD05-T1	0.58	0.18	0.03	0.16	0.04	0.25	0.04	0.13	0.03	0.22	0.03	4.69		3.79	0.9	4.21		2.9	0.53	1.09	[13]
16	LD05-T2	0.15	0.06	0.01	0.07	0.02	0.11	0.02	0.05	0.01	0.08	0.01	1.54		1.17	0.37	3.16		2.06	0.47	1.46	
17	LD05-T3	0.99	0.36	0.03	0.36	0.09	0.55	0.09	0.26	0.05	0.39	0.06	7.95		6.1	1.85	3.3		2.34	0.25	1.19	
18	LD10-T1	0.69	0.19	0.09	0.17	0.03	0.17	0.03	0.1	0.02	0.11	0.02	4.89		4.24	0.65	6.52		5.93	1.5	1.21	
19	LD10-T2	2.85	0.71	0.26	0.62	0.11	0.59	0.11	0.32	0.05	0.31	0.04	17.11		14.96	2.15	6.96		7.89	1.17	1.02	
20	LD10-T3	0.21	0.07	0.07	0.13	0.03	0.23	0.05	0.17	0.03	0.19	0.03	3.08		2.22	0.86	2.58		1.77	2.21	1.67	
21	SP01*	2.83	0.93	0.42	1.26	0.22	1.25	0.19	0.48	0.06	0.33	0.05	17.7		13.86	3.84	3.61		6.52	1.19	0.99	[20]
22	SP02*	1.29	0.43	0.47	0.55	0.09	0.55	0.09	0.22	0.03	0.15	0.02	11.65		9.95	1.7	5.85		7.94	2.95	1.8	
23	PD9851	0.9	0.41	0.63	0.48	0.18	0.96	0.16	0.38	0.06	0.23	0.03	8.12		5.64	2.48	2.27		4.68	4.33	0.77	[16]
24	PD10353	5.7	1.84	1.23	2.1	0.61	3.86	0.79	2.14	0.33	1.63	0.22	38.75		27.07	11.68	2.32		2.6	1.91	0.91	
25	PD10804	3.9	1.42	0.49	1.91	0.53	3.26	0.63	1.54	0.19	0.94	0.12	28.73		19.61	9.12	2.15		3.43	0.91	0.92	
26	PD9454	2.5	0.62	0.3	0.72	0.19	1.06	0.21	0.48	0.09	0.46	0.06	17.99		14.72	3.27	4.5		6.08	1.37	0.92	
27	PD98511	4.0	0.98	1.29	1.26	0.32	2.58	0.55	1.41	0.25	1.48	0.17	33.99		25.97	8.02	3.24		3.34	3.55	0.91	
28	3GSG40	1.6	0.37	0.34	0.33	0.05	0.30	0.04	0.10	0.01	0.07	0.01	11.42		10.51	0.91	11.55		28.69	2.91	0.94	
29	XHC-14	63.6	17.6	0.42	18.8	4.3	26.2	5.36	13.5	2.22	11.6	1.34	396.84		313.52	83.32	3.76		4.36	0.07	0.98	[19]
30	XHC-6(2)	2.84	0.83	0.29	0.99	0.31	2.24	0.43	1.3	0.23	1.68	0.22	24.04		16.64	7.4	2.25		1.87	0.98	0.9	
31	PD945−9	92.7	19.5	1.08	16	2.74	11.4	1.7	3.32	0.38	1.54	0.18	513.94		476.68	37.26	12.79		52.63	0.18	0.99	
32	B1	0.230	0.086	0.020	0.052	0.011	0.062	0.011	0.026	0.005	0.039	0.005	1.93		1.72	0.21	8.17		9.38	0.85	0.88	[23]
33	B2	0.180	0.088	0.015	0.041	0.007	0.055	0.008	0.028	0.005	0.041	0.003	1.39		1.2	0.19	6.4		5.77	0.67	0.91	
34	B3	0.230	0.100	0.021	0.063	0.011	0.079	0.014	0.042	0.004	0.044	0.006	1.81		1.55	0.26	5.87		7.5	0.75	0.84	
35	B4	0.18	0.079	0.018	0.041	0.006	0.039	0.007	0.023	0.005	0.029	0.003	1.3		1.15	0.15	7.48		7.92	0.87	0.88	
36	B5	1.960	1.087	0.042	1.291	0.400	1.612	0.156	0.328	0.053	0.329	0.037	14.94		10.73	4.21	2.55		4.75	0.11	1.06	
37	B6	6.240	1.031	0.247	1.160	0.153	0.799	0.132	0.335	0.045	0.292	0.035	23.14		20.19	2.95	6.84		22.26	0.69	0.11	
38	B7	0.990	0.363	0.114	0.350	0.099	0.435	0.049	0.107	0.017	0.127	0.018	6.37		5.17	1.2	4.3		7.29	0.96	0.81	
39	B8	0.620	0.460	0.028	0.662	0.245	0.868	0.073	0.113	0.014	0.083	0.011	5.8		3.73	2.07	1.8		6.74	0.16	1.03	
40	B9	0.700	0.176	0.034	0.137	0.018	0.107	0.023	0.059	0.008	0.050	0.012	3.15		2.73	0.41	6.6		15.49	0.65	0.27	
41	2	10.9	5.34	0.21	8.03	2.27	19.5	4.94	20.2	4.7	43.2	8.32	160.25		49.09	111.16	0.44		0.14	0.1	1.1	[21]
42	3	7.89	2.68	0.1	3.47	0.9	6.64	1.36	4.76	0.91	7.91	1.22	66.79		39.62	27.17	1.46		0.64	0.1	1.22	
43	4	2.81	0.95	0.13	1.24	0.29	1.88	0.36	1.21	0.3	2.37	0.38	22.71		14.68	8.03	1.83		0.92	0.37	0.98	
44	5	7.05	2.1l	0.21	2.28	0.55	4.98	1.2	4.68	1	10.8	1.93	67.4		39.98	27.42	1.46		0.45	0.29	1.5	
45	6	5.22	2.15	0.1	2.63	0.64	4.29	0.94	3.4	0.71	5.96	0.85	47.24		27.82	19.42	1.43		0.61	0.13	1.26	

Note: ΣREE = total rare earth elements (La–Lu + Y); LREE = light REE (La–Eu); HREE = heavy REE (Gd–Lu + Y); LREE/HREE = concentration ratio; La_N_/Yb_N_ = chondrite-normalized ratio; δEu = Eu_N_/(Sm_N_ × Gd_N_); δCe = CeN/(LaN × PrN)”

## 6. Discussion

### 6.1. Magmatic differentiation and geochemical evolution

Previous studies have suggested a genetic relationship between the Caledonian Huichizi pluton and the surrounding granitic pegmatites, indicating that the pluton serves as the parent rock for these pegmatites [14,24]. The spatial distribution of pegmatites is controlled by the associated granite, forming concentric zonation around the parent pluton [18]. The types of pegmatites systematically evolve from biotite–microcline pegmatites to muscovite (or lepidolite) albite pegmatites, with this spatial transition also reflecting temporal evolution to some extent [18,24]. This spatial and temporal evolution results in distinct variations in the chemical compositions of different pegmatite types. For instance, uranium–mineralized biotite–microcline pegmatites proximal to the Huichizi pluton have higher Fe, Mg, and Ti contents and lower Na content (Figs 7b, 7e). This composition reflects their early crystallization stage, where compatible elements like Fe and Mg remained in the melt, and extensive fluid–rock interaction with surrounding country rocks may have contributed additional Fe–Mg components. The lower Na content signifies limited plagioclase fractionation and a less evolved melt at this stage.

In contrast, distal lithium–beryllium–mineralized and HPQ–mineralized muscovite–albite pegmatites exhibit significantly lower Fe, Mg, and Ti contents but higher Na concentrations. The depletion in Fe, Mg, and Ti is a classic indicator of extreme fractional crystallization, where these elements were sequestered in early–forming ferromagnesian minerals such as biotite and accessory phases like ilmenite. Conversely, the pronounced Na enrichment signifies protracted fractionation leading to an albite–rich residual melt, often accompanied by intense alkali metasomatism (albitization) which is critical for both rare–metal enrichment and quartz purification. This compositional differentiation aligns with mineralization zoning: pegmatites near the parent pluton are predominantly uranium–mineralized, such as the Guangshigou and Xiaohuacha uranium pegmatites, while distal pegmatites host Li–Be–Nb–Ta mineralization, as seen in the Guanpo rare–metal pegmatites and Fenghuangzhai lithium deposits. Notably, the Shangnan HPQ pegmatites are located approximately 10 km from the Huichizi pluton, while the Longquanping LD5 HPQ pegmatite is about 8 km away, placing them in an “intermediate” zone spatially between uranium mineralized and rare metal mineralized pegmatites [9].

Exploration in the research area and neighboring regions reveals that pegmatites capable of producing quartz purified to >99.99% SiO_2_ are primarily muscovite–albite pegmatites. Geochemical analyses indicate that the Shangnan pegmatites consistently have higher Na contents than K, with K_2_O/Na_2_O ratios ranging from 0.2 to 1.09. This K_2_O/Na_2_O ratio of less than 1 is a key discriminant. It signals a melt that evolved beyond the microcline stability field into the albite–dominant regime. Such evolution is conducive to the exclusion of alkali impurities (especially K) from the quartz lattice and is often associated with late–stage, low–temperature, Na–rich hydrothermal fluids that can leach impurities from pre–existing quartz. This Na enrichment is even more pronounced in the Spruce–Pine pegmatites, where K_2_O/Na_2_O ratios range from 0.19 to 0.52, suggesting that muscovite–albite pegmatites are the most favorable type for HPQ mineralization. The parallel between the study area and Spruce–Pine underscores a fundamental petrogenetic rule: the formation of HPQ is not accidental but is intrinsically linked to a specific, advanced stage of peraluminous granite–pegmatite differentiation characterized by strong sodic alteration.

### 6.2. High differentiation affects quartz purification

As the pegmatites evolve from less to more differentiated types [14], their degree of differentiation increases significantly [24]. Among the three types of pegmatites associated with the Huichizi pluton, differentiation indices (DI) vary notably. Uranium–mineralized pegmatites near the pluton exhibit the lowest differentiation (DI: 82.8–94.0), followed by Li–Be mineralized pegmatites (DI: 83.8–95.2), and HPQ pegmatites exhibit the highest differentiation (DI: 89.7–98.4). The exceptionally high DI values in the HPQ pegmatites indicate an extreme depletion of mafic components and concentration of silica and alkalies in the final residual melts/fluids. This level of differentiation is a prerequisite for generating quartz with minimal intracrystalline impurities like Al, Ti, and Ge, which commonly substitute for Si in less evolved systems. This differentiation degree is reflected in the internal zoning characteristics: uranium–mineralized pegmatites often lack clear zoning, rare–metal mineralized pegmatites display distinct zoning, and highly differentiated HPQ pegmatites exhibit well–developed zonation.

In HPQ pegmatites, this pronounced zonation has a minimal impact on quartz impurity levels and purification outcomes. For example, quartz from the coarse–grained and massive zones demonstrated marginally better purification results, while zones near the pegmatite margins, likely affected by wall–rock contamination, contained higher impurities. This implies that the intrinsic purity of the quartz is primarily inherited from the composition of the crystallizing melt/fluid, with wall–rock contamination being a secondary, localized overprint. The homogeneity of HPQ across different internal zones, except at the margins, underscores the effectiveness of the magmatic–hydrothermal system in self–purification. These findings suggest that the potential for HPQ formation is more dependent on the highly evolved composition of the granitic magma rather than structural zoning during emplacement and crystallization. Therefore, the search for HPQ should prioritize identifying the most highly fractionated pegmatite bodies, as indicated by geochemical proxies like low K/Na, low Fe + Mg + Ti, and high DI, rather than solely relying on structural complexity.

Metamorphism may also contribute to quartz purification. Müller et al. (2012) have demonstrated that Li concentrations in quartz decrease during recrystallization, and Al concentrations may also reduce under specific conditions [2]. Additionally, Götze et al. (2017) proposed that metamorphism–driven quartz recrystallization, defect healing, and the breakdown of inclusions can lead to reduced trace element concentrations, facilitating natural “purification” [25]. This post–magmatic process could be particularly relevant for ancient pegmatites like those in the East Qinling. Dynamic recrystallization under greenschist to amphibolite facies conditions can expel impurities from the quartz lattice by facilitating diffusion and recovery processes, thereby enhancing the final purity attainable through industrial processing. Geochronological studies indicate that pegmatites in the region formed during the Silurian (ca. 420 Ma). For instance, the Guangshigou–Xiaohuacha biotite pegmatites were dated to 405–418 Ma [17,23], and the Longquanping muscovite–albite pegmatites were dated to 406–420 Ma [13,21]. These pegmatites likely experienced multiple episodes of deformation and metamorphism post–emplacement, such as those occurring between 370–320 Ma [26,27]). The structural control of the gneiss domes and potential influence of deep–seated plutons or metamorphic events likely played a significant role in concentrating HPQ pegmatites in the research area.

### 6.3. Regional comparison and exploration significance

Globally, pegmatites capable of producing HPQ are rare [2,19]. The geological setting, petrological characteristics, and geochemical features of the research area’s pegmatites are strikingly similar to those of the renowned Spruce–Pine pegmatites. Both are situated within deformed Precambrian basement terrains intruded by later granitic plutons, both exhibit clear concentric zoning of pegmatite types around parent granites, and both host HPQ in highly fractionated, sodic (albite–rich) pegmatites [21]. This analogy is not merely descriptive but genetic, suggesting comparable tectonic settings (post–orogenic extension/crustal melting) and similar deep magmatic processes leading to extreme differentiation [28].

The discoveries of HPQ pegmatites in the Longquanping region and the current research area highlight the region’s tremendous exploration potential for this valuable resource. The established spatial–compositional–mineralizational model around the Huichizi pluton provides a powerful exploration vector. Future prospecting should focus on the distal zones (approximately 8–15 km) from similar Caledonian parent plutons within the East Qinling, targeting muscovite–albite pegmatites with geochemical signatures of extreme differentiation (high DI, low K/Na, low Fe + Mg + Ti). Furthermore, areas within regional metamorphic domes or shear zones, where post–magmatic recrystallization may have occurred, could be prioritized as these processes might enhance quartz purity. This study, therefore, not only deciphers the origin of a specific deposit but also establishes a replicable exploration framework that could significantly contribute to securing a domestic supply of this critical strategic mineral.

## 7. Conclusion

(1) High–purity quartz deposits of the pegmatite–type have been discovered in the Shangnan of Shaanxi Province, China. The pegmatite veins are classified as two–mica (microcline) albite pegmatites and exhibit well–developed structural zonation. Preliminary purification of quartz from large veins has achieved SiO_2_ purities exceeding 99.99%, with a maximum purity of 99.997%.
(2) High–purity quartz pegmatites are classified as peraluminous alkaline granites and exhibit significant geochemical distinctions from uranium mineralized and rare–metal mineralized pegmatites in the region. They are characterized by low Fe and Mg contents, high Na content, and pronounced enrichment in U, P, and Hf, while showing obviously depletion in Ba and Ti. The REE content is extremely low, with flat chondrite–normalized REE patterns and negligible fractionation between light and heavy REEs.

The type and geochemical characteristics of pegmatites are closely related to their temporal and spatial distribution. High–purity quartz pegmatites are products of late–stage, highly evolved differentiation of pegmatitic systems. Na–rich pegmatites closely resemble the Spruce–Pine pegmatites, making them the most favorable type for high–purity quartz mineralization. The purity of quartz in these pegmatites is likely determined primarily by the composition of highly evolved granitic magmas formed during magmatic differentiation, while subsequent metamorphic and deformational processes contribute to quartz “purification.”

## Supporting information

S1 File XRF and ICP-MS analytical quality control data of certified reference materials (CRMs). (XLSX)

S2 File Calibration curve parameters of major elements determined by XRF spectrometry. (XLS)
